# Warming-dredging acupuncture for cancer-related pain: a systematic review and meta-analysis

**DOI:** 10.3389/fpain.2026.1870255

**Published:** 2026-07-30

**Authors:** Jianlong Tu, Qiumei Wu, Jing Wu, Lunling Gan, Jing Xu, Xiaoting Xu

**Affiliations:** 1 Department of Oncology, Huizhou Hospital of Traditional Chinese Medicine (Huizhou Hospital of Guangzhou University of Chinese Medicine), Huizhou, Guangdong, China; 2 Graduate School, Guangzhou University of Chinese Medicine, Guangzhou, Guangdong, China; 3 Clinical Department, Longbio Pharma (Suzhou) Co., Ltd., Suzhou, Jiangsu, China; 4 Outpatient Department, Tian'an Cloud Park Community Health Service Center, Longgang District People’s Hospital, Shenzhen, Guangdong, China

**Keywords:** cancer-related pain, fire needle, meta-analysis, opioid-sparing effect, systematic review, warm needle acupuncture, warming-dredging acupuncture

## Abstract

**Background/objectives:**

Cancer-related pain (CRP) affects up to 60% of patients during malignancy progression, with many experiencing insufficient relief from pharmacological treatments alone. Warming-dredging acupuncture (WDA) integrates thermal and mechanical stimulation to achieve synergistic analgesic effects. This study aims to evaluate the efficacy, safety, and opioid-sparing potential of WDA in managing CRP.

**Materials and methods:**

We searched PubMed/MEDLINE, Embase, CENTRAL, Web of Science, CNKI, VIP, Wanfang Data, SinoMed, and the clinical trial registries (ClinicalTrials.gov and the Chinese Clinical Trial Registry) through March 9, 2026. Only randomized controlled trials (RCTs) were included. We used RevMan 5.4 and Stata 19.0 to analyze data, the RoB 2 tool to assess the risk of bias, and the GRADE system to assess the certainty of the evidence, following the PRISMA 2020 guidelines. The protocol was prospectively registered (PROSPERO: CRD420261335674).

**Results:**

Seventeen RCTs involving 1278 participants were included. WDA significantly enhanced pain relief rates (RR = 1.26; 95% CI: 1.19–1.33) and reduced pain scores (MD = −1.05; 95% CI: −1.49 to −0.61) compared to controls. WDA also showed a lower risk of adverse events (RR = 0.35; 95% CI: 0.26–0.46), and evidence suggested a possible reduction trend in analgesic consumption. GRADE certainty ranged from moderate to very low.

**Conclusions:**

Warming-dredging acupuncture combined with routine pain management may offer a promising adjunct for multimodal cancer pain management, but the certainty of evidence varied across outcomes.

**Systematic Review Registration:**

https://www.crd.york.ac.uk/PROSPERO/view/CRD420261335674 identifier CRD420261335674.

## 1 Introduction

Cancer-related pain (CRP) remains a widespread problem for patients with cancer. Recent studies (1) show that 45%–60% of patients with tumors report suffering from pain. With malignancy progressing and the treatment course increasing, the rate of CRP rises to nearly 60% (2). Among patients with CRP, one-third have moderate-to-severe cancer pain (1), and even 34% to 49% of tumor survivors (3) endure chronic CRP. In the last year of life (4), the proportion increases, reaching 82% in the final week (5). Since 1986, the WHO has recommended the three-step analgesic ladder therapy as the standard treatment for CRP; however, the side effects of drugs such as nausea, constipation, and sedation are increasingly unbearable for patients as the dosages increase (6, 7). Also, the economic burden remains a problem. Patients need to incur an additional 66% in costs compared to those without CRP (8). Despite all these limitations, about 30% of patients suffering from CRP are still receiving inadequate pain relief from drugs alone (9, 10). In recent years, the guidelines have begun to suggest that clinicians choose multimodal strategies for pain management, especially emphasizing the integration of non-pharmacological interventions (11–13).

Acupuncture is widely known as an effective treatment for CRP (14). In traditional methods, all effects rely on achieving the goal of Deqi (15) through various forms of manual manipulation, which requires the practitioner's personal experience. So, the therapeutic effects are quite variable and individualized (16). Which manipulation of acupuncture is more effective, as well as how to achieve Deqi more easily, remains a question. To address these, researchers introduced “warming-dredging” acupuncture (17–19) as an effective clinical choice. In this review, warming-dredging acupuncture is operationally defined as a category of invasive thermal acupuncture techniques that combine needle puncture, insertion, or retention with heat delivered by or through the needle itself. This technique exerts its therapeutic effects through the analgesic action resulting from the combination of thermal and mechanical stimulation. In practice, warming-dredging acupuncture usually takes the form of fire needle (FN) or warm needle acupuncture (WNA). This warming-dredging concept holds that thermal energy is a complementary intervention variable, alongside needles’ mechanical stimulation, that would bring a better therapeutic effect (17). In traditional Chinese medicine, pain is commonly considered to result from obstruction of qi and blood circulation. Conventional acupuncture regulates qi and blood circulation mainly through mechanical stimulation, whereas warming-dredging acupuncture further introduces needle-mediated thermal stimulation. It aims to warm the meridians, dredge the obstruction, and relieve the pain by combining mechanical and thermal stimulation (20). Thermal energy input provides a more objective and easier method to elicit Deqi than individual manual techniques. From a modern mechanistic perspective, heat activates local temperature-sensitive ion channels (21, 22) such as Transient Receptor Potential Vanilloid 1 (TRPV1), and mechanical stimulation triggers mechanoreceptors and their associated signaling pathways to reduce pain. Both thermal and mechanical stimulation can reduce pain; combined stimulation can minimize the dependence on individual manual techniques, resulting in a synergistic effect (23).

Prior meta-analyses focus more on acupuncture in general than on warming-dredging acupuncture for CRP; warming-dredging acupuncture usually appears as a subgroup treatment (24). The clinical trials are often small-cohort and yield inconsistent effect estimates (25). The evidence is fragmented. This lack of consolidated evidence leads to unclear guidance for clinicians to choose this method for CRP. To date, no systematic review has evaluated the collective evidence for warming-dredging acupuncture in CRP. To solve this problem, we conducted this systematic review and meta-analysis. We integrated evidence on the “warming-dredging” acupuncture to evaluate its effect on CRP and safety profile. Moreover, we investigated the effect on opioid-sparing potential. The impact on functional status measured by the Karnofsky Performance Status (KPS) and quality of life (QoL) was also assessed.

## 2 Methods

### 2.1 Study design and registration

This systematic review and meta-analysis was strictly designed and conducted following the guidelines of the Preferred Reporting Items for Systematic Reviews and Meta-Analyses (PRISMA 2020) (26). This protocol has already been registered on the Prospective Register of Systematic Reviews (PROSPERO). The registration number was CRD420261335674 (available from https://www.crd.york.ac.uk/PROSPERO/view/CRD420261335674).

### 2.2 Literature search strategy and study selection

We searched the literature through 8 electronic bibliographic sources: PubMed/MEDLINE, Embase, the Cochrane Library including the Cochrane Central Register of Controlled Trials (CENTRAL), Web of Science, CNKI, VIP, Wanfang Data, and SinoMed. To obtain enough trials, we searched the clinical trial registries as well: ClinicalTrials.gov and the Chinese Clinical Trial Registry (ChiCTR). We did not set any restrictions on the language. The literature published on each database from inception to March 9, 2026, was eligible.

We used the controlled vocabulary (e.g., MeSH and Emtree terms) combined with free-text keywords as the search strategy. We focused on the three primary domains: warming-dredging acupuncture as the intervention, cancer-related pain terms as the target population, and randomized controlled trials as the study design. All detailed search strings of each database were outlined in Supplementary Table S1.

### 2.3 Inclusion criteria

According to the PICOS (Population, Intervention, Comparison, Outcome, and Study Design) framework, we included studies by the following criteria:

Population (P): Patients were diagnosed with any kind of malignancy and suffered from CRP. We did not set any restrictions on age, sex, or cancer stage.

Intervention (I): Warming-dredging acupuncture was defined as invasive needle-based thermal acupuncture combining needle puncture, insertion, or retention with heat delivered by or through the needle itself. Routine pain care management was allowed as a concomitant treatment.

Comparison (C): Standard pain treatments (e.g., analgesia or other active controls) were set as the comparison method. The comparison treatments should provide the same basic treatment as the intervention group.

Outcome (O): Pain relief rate and pain intensity measured by the Visual Analogue Scale (VAS) or the Numerical Rating Scale (NRS) were the primary outcomes. Secondary outcomes covered analgesic dosages, QoL scores, physical status measured by the KPS, and adverse events.

Study Design (S): Only randomized controlled trials (RCTs) were eligible.

### 2.4 Exclusion criteria

Studies were excluded by the following criteria:

1. Animal studies, conference reports, or other brief reports lacking sufficient details.
2. Interventions lacking either needle insertion or needle-mediated thermal stimulation were excluded, such as moxibustion or acupoint heat application (without needling), and conventional acupuncture (without heat).
3. Treatment group and control group received different background pain management (e.g., warming-dredging acupuncture alone vs. opioid).
4. Insufficient Data: Trials with insufficient data for primary outcomes.

### 2.5 Data extraction

Two authors, Qiumei Wu and Jing Wu, independently conducted data extraction. Disagreements were resolved through discussions or adjudicated by a third author, Jianlong Tu. Study characteristics were collected into an Excel table, including: first author, publication year, country, patient demographics (total sample size, mean age, sex distribution, malignancy type, and baseline pain severity), intervention and control protocols (acupuncture technique, acupoint selection, and treatment duration), study outcomes, and the adverse events. After that, we extracted the relevant numerical values into a standardized Excel spreadsheet for the following analysis.

### 2.6 Risk of bias and quality assessment

To assess the risk of bias for the included studies, we used the Cochrane RoB 2 tool (27). We assessed the five domains: randomization process, deviations from intended interventions, missing outcome data, outcome measurement, and selection of the reported result; each domain was evaluated as “low risk,” “some concerns,” or “high risk.” According to the domain assessments, an overall assessment of each trial was rated as the same three grades. Plots of the risk of bias assessments were generated using *robvis* (28) (available from https://mcguinlu.shinyapps.io/robvis/). To rate the certainty of evidence for outcomes, we employed the Grading of Recommendations, Assessment, Development, and Evaluation (GRADE) framework (29). According to the five domains (risk of bias, inconsistency, indirectness, imprecision, and publication bias), we rated each outcome as four grades (high, moderate, low, or very low). Two authors, Lunling Gan and Jing Xu, completed this assessment part independently. Any discrepancies were resolved by discussion or consultation with a third author, Jianlong Tu.

### 2.7 Statistical analysis

We used RevMan (version 5.4) and Stata (version 19.0) for data analysis, and all figures were visualized by Stata 19.0 except risk of bias assessment plots. We used risk ratios (RRs) with 95% confidence intervals (CIs) to analyze the dichotomous data and mean differences (MDs) with 95% CIs to analyze the continuous data. Pain intensity measured by VAS and NRS was pooled using MD because both instruments assess pain intensity on comparable 0–10 scales. As some continuous data with significant heterogeneity could not be pooled together for meta-analysis, we employed qualitative description combined with percentage change to display the results. The percentage change is calculated using the following method:

$$\mathrm{Percentage}\;\mathrm{Change}=\frac{\mathrm{Mea}n_{\mathrm{Control}}-\mathrm{Mea}n_{\mathrm{Intervention}}}{\mathrm{Mea}n_{\mathrm{Control}}}\times 100$$

We used the Chi-square test to assess whether heterogeneity existed and used *I*^2^ to quantify it. *I*² values of approximately 25%, 50%, and 75% were considered to indicate low, moderate, and high heterogeneity, respectively, according to Higgins criteria (30). And following the Cochrane guidelines, a random-effects model was used to analyze when the *I*^2^ values were >50%, indicating moderate-to-high heterogeneity. A fixed-effects model was used to analyze when the *I*^2^ values were ≤50%. Subgroup analyses were conducted to explore potential sources of heterogeneity by following grouping: modalities (fire needle vs. warm needle acupuncture) and fire needle types (conventional fire needle vs. filiform-fire needle) as described in the PROSPERO registration, and treatment duration (long-term: >2 weeks vs. short-term: ≤2 weeks) as a *post hoc* exploratory analysis. A two-tailed *P* value less than 0.05 was defined as statistically significant, except for the heterogeneity test (*α* = 0.1).

We also conducted several sensitivity analyses to verify the robustness of our findings. We divided all included trials into two subgroups, gray literature and peer-reviewed literature, to test whether unpublished data had a material influence on the results. Then we performed a leave-one-out analysis to assess whether there was one trial exerting a decisive influence on the overall effect sizes and to explore the sources of heterogeneity. When high heterogeneity existed, we applied Galbraith (radial) plots to identify outliers, combined with the leave-one-out analysis, to find whether specific trials drove the results.

For outcomes including at least 10 trials, we assessed the publication bias through the funnel plot by visual symmetry and Egger's test. When we found evidence that bias existed, we used Stata 19.0 to perform a trim-and-fill method to test whether potential small-study effects would alter the conclusions.

## 3 Results

### 3.1 Study identification

**Figure 1:**
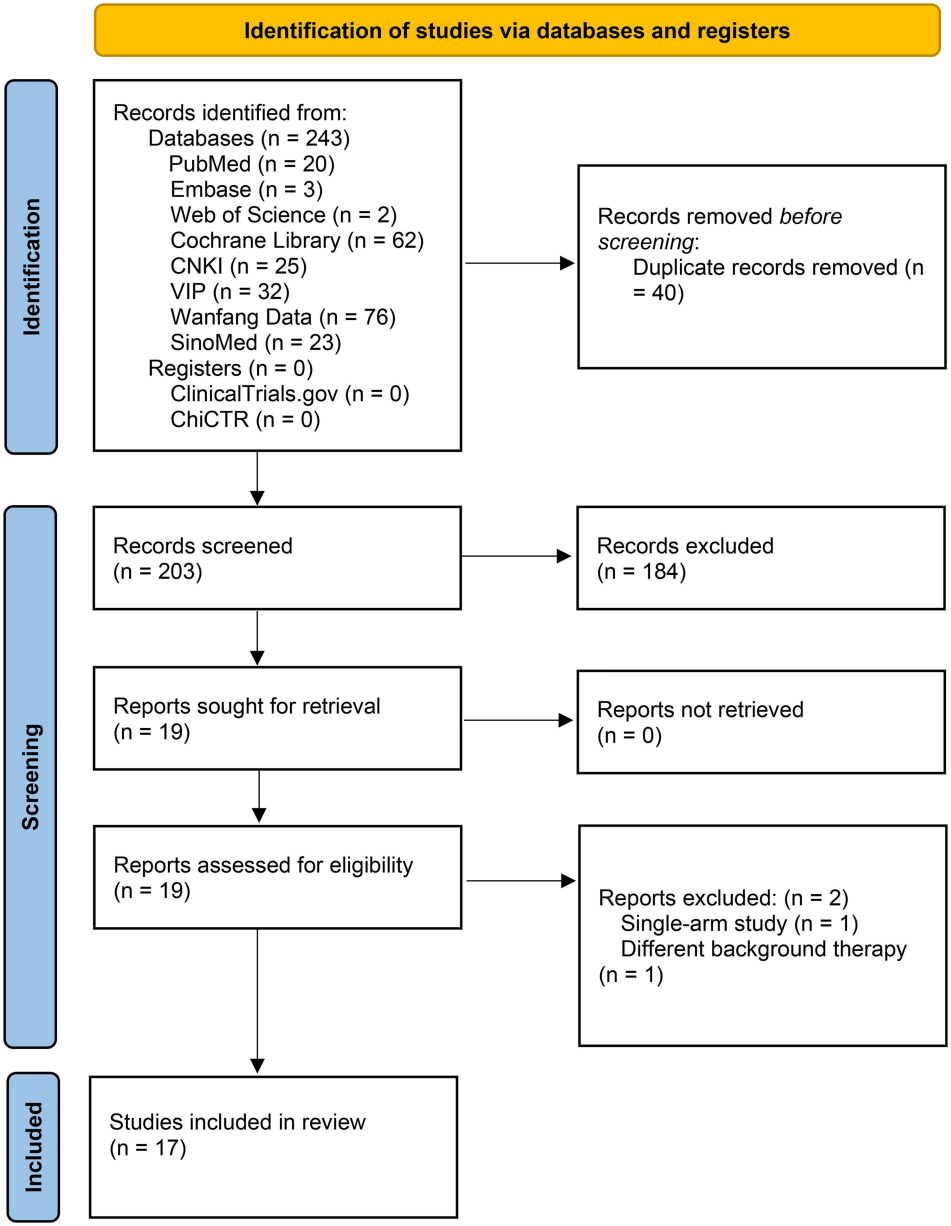
PRISMA 2020 flow diagram of the study selection process.

We found 243 records after initial searching. After using EndNote 2025 to remove 40 duplicates, 203 records were left. We then excluded 184 records by screening titles and abstracts that did not meet the eligibility criteria. All full texts of the 19 remaining studies were evaluated, and one single-arm study and one trial comparing fire needle acupuncture alone with opioids were excluded. Finally, 17 RCTs (31–47) met all criteria for analysis (process shown in Figure 1).

### 3.2 Study characteristics

All clinical characteristics of the 17 included trials involving 1,278 participants were detailed in Table 1. @ (31–47) All 17 studies were conducted in China, and 4 papers (34, 39–41) were master's theses. Only 1 trial (41) did not report the age distribution; the remaining 16 studies showed a mean age of 54.3 ± 11.1 years. Ten studies (31–34, 39–43, 45) reported baseline pain intensity (measured by VAS or NRS), with a mean score of 6.71 ± 1.62 points. Except that 1 trial (33) focused on cervical cancer and 1 trial (36) did not report sex distribution, 15 studies reported mixed sex population compositions with male proportions ranging from 35.5% to 78.3%. Cancer types varied: 6 studies (33, 34, 37, 38, 40, 44) focused on a single type, 8 included mixed cancer types (31, 32, 35, 39, 41–43, 45), and 3 were unspecified (36, 46, 47).

**Table 1:** Summary characteristics of the 17 included trials.

Study (Year)	Publication type	Sample Size (T/C)	Age	Male (%)	Cancer Type	Intervention Protocol	Pain Scores baseline	Duration
Bai et al., 2019 (31)	Journal article	100 (50/50)	47.00 ± 8.43	54 (54.0%)	Mixed	Filiform-fire needle + routine care	8.20 ± 1.28	2 weeks
Gao et al., 2020 (32)	Journal article	60 (30/30)	53.5 ± 8.0	31 (51.7%)	Mixed	Filiform-fire needle + routine care	7.14 ± 1.81	4 weeks
Gao et al., 2024 (33)	Journal article	84 (42/42)	47.77 ± 6.05	0 (0%)	Cervical	Warming needle + routine care	5.83 ± 1.10	7 days
Hu 2020 (34)	Master’s thesis	60 (30/30)	54.50 ± 12.31	47 (78.3%)	Liver	Warming needle + routine care	5.72 ± 0.46	4 weeks
Huang et al., 2020 (35)	Journal article	98 (49/49)	47.27 ± 2.38	54 (55.1%)	Mixed	Warming needle + routine care	NR	6 days
Lai et al., 2011 (36)	Journal article	70 (35/35)*	59.30 ± 12.10	NR	NR	Warming needle + routine care	NR	10 days
Mi et al., 2010 (37)	Journal article	62 (32/30)	44.00 ± 10.60	22 (35.5%)	Gastric	Fire needle + routine care	NR	4 weeks
Song 2025 (39)	Master’s thesis	60 (30/30)	63.44 ± 8.62	35 (58.3%)	Mixed	Fire needle + routine care	5.25 ± 0.74	2 weeks
Song et al. 2025(38)	Journal article	90 (45/45)	49.83 ± 5.95	48 (53.3%)	Lung	Warming needle + routine care	NR	7 days
Wang 2019 (40)	Master’s thesis	40 (20/20)*	59.30 ± 10.41	18 (45.0%)	Bone	Warming needle + routine care	8.09 ± 1.68	4 weeks
Wang 2022 (41)	Master's thesis	78 (39/39)	NR	53 (67.9%)	Mixed	Filiform-fire needle + routine care	5.00 ± 1.48	2 weeks
Wei et al., 2025 (42)	Journal article	60 (30/30)	67.14 ± 6.82	25 (41.7%)	Mixed	Warming needle + routine care	6.38 ± 1.49	2 weeks
Yao et al., 2021 (43)	Journal article	144 (72/72)	67.88 ± 2.85	74 (51.4%)	Mixed	Warming needle + routine care	7.30 ± 0.20	4 weeks
Ying et al., 2017 (44)	Journal article	50 (25/25)	50.05 ± 8.12	39 (78%)	Liver	Fire needle + routine care	NR	2 weeks
Yuan et al., 2022 (45)	Journal article	80 (40/40)	52.68 ± 5.47	47 (58.8%)	Mixed	Filiform-fire needle + routine care	7.47 ± 1.56	2 weeks
Zang and Zhao, 2018 (46)	Journal article	80 (40/40)*	44.00 ± 3.31	53 (56.3%)	NR	Fire needle + routine care	NR	10 days
Zhang and Ni, 2020 (47)	Journal article	62 (31/31)	60.48 ± 10.63	24 (38.7%)	NR	Fire needle + routine care	NR	4 weeks

T, treatment; C, control; NR, not reported.

* The third arm (e.g., Electro-acupuncture) was excluded to maintain consistency with the intervention criteria. All 17 trials were conducted in China; all control groups received routine care.

Regarding intervention, 8 trials (33–36, 38, 40, 42, 43) applied warm needle acupuncture while 9 applied fire needle acupuncture (31, 32, 37, 39, 41, 44–47) (5 using conventional fire needles (37, 39, 44, 46, 47) and 4 using filiform fire needles (31, 32, 41, 45) with routine pain management. For 3 trials (36, 40, 46) with a three-arm design, we extracted data for the relevant two-group design as a single trial. Treatment durations ranged from 6 days to 4 weeks. Acupoints were detailed in Supplementary Table S2, and we identified Zusanli (ST36, 58.8%), Ashi points (35.3%), Hegu (LI4, 35.3%), and Guanyuan (CV4, 35.3%) as the most frequently utilized points.

### 3.3 Risk of bias

**Figure 2:**
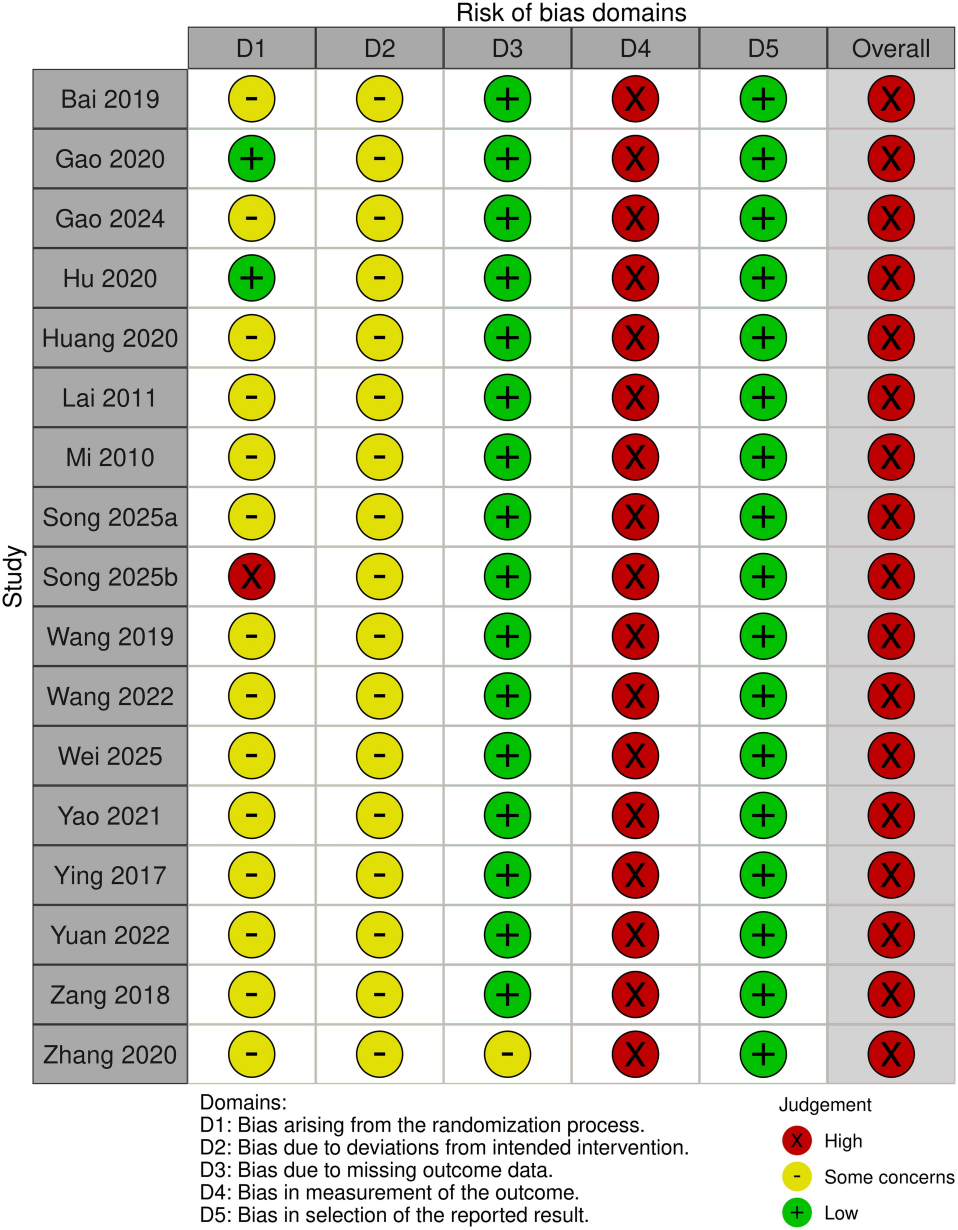
Traffic light plot for the risk of bias of all included studies.

The overall risk of bias for the 17 included RCTs was assessed as high risk due to the inevitable absence of blinding and the subjective measurements for outcomes by using the RoB 2 tool (shown in Figure 2 and Supplementary Figure S1).

Randomization process (D1): The majority of studies (*n* = 16) used random number tables or computer-generated sequences for randomization; however, 14 of them did not mention the details of allocations that were assessed as some concerns, while two studies (32, 34) were rated as low risk due to the specific description of allocation concealment. Conversely, one study (38) was rated as high risk due to failing to provide randomization details.

Deviations from intended interventions (D2): Warming-dredging acupuncture requires physical and thermal stimulation, making blinding difficult for both participants and practitioners. Since the grouping was known and may have influenced the results, all studies were rated as some concerns in the D2 domain.

Missing outcome data (D3): one trial (47) was rated as some concerns because the study reported that 8 participants (11.4%) dropped out without providing details. The other trials were rated as low risk because of complete outcome data or a dropout rate of less than five percent.

Measurement of the outcome (D4): All primary outcomes were subjective and measured in an unblinded setting. Thus, the results were easily influenced. All 17 RCTs were rated as high risk.

Selection of the reported results (D5): All 17 studies were rated as low risk because they reported outcomes consistent with the prespecified methodological objectives.

### 3.4 Primary outcomes

#### 3.4.1 Pain relief rate and pain score changes

**Figure 3:**
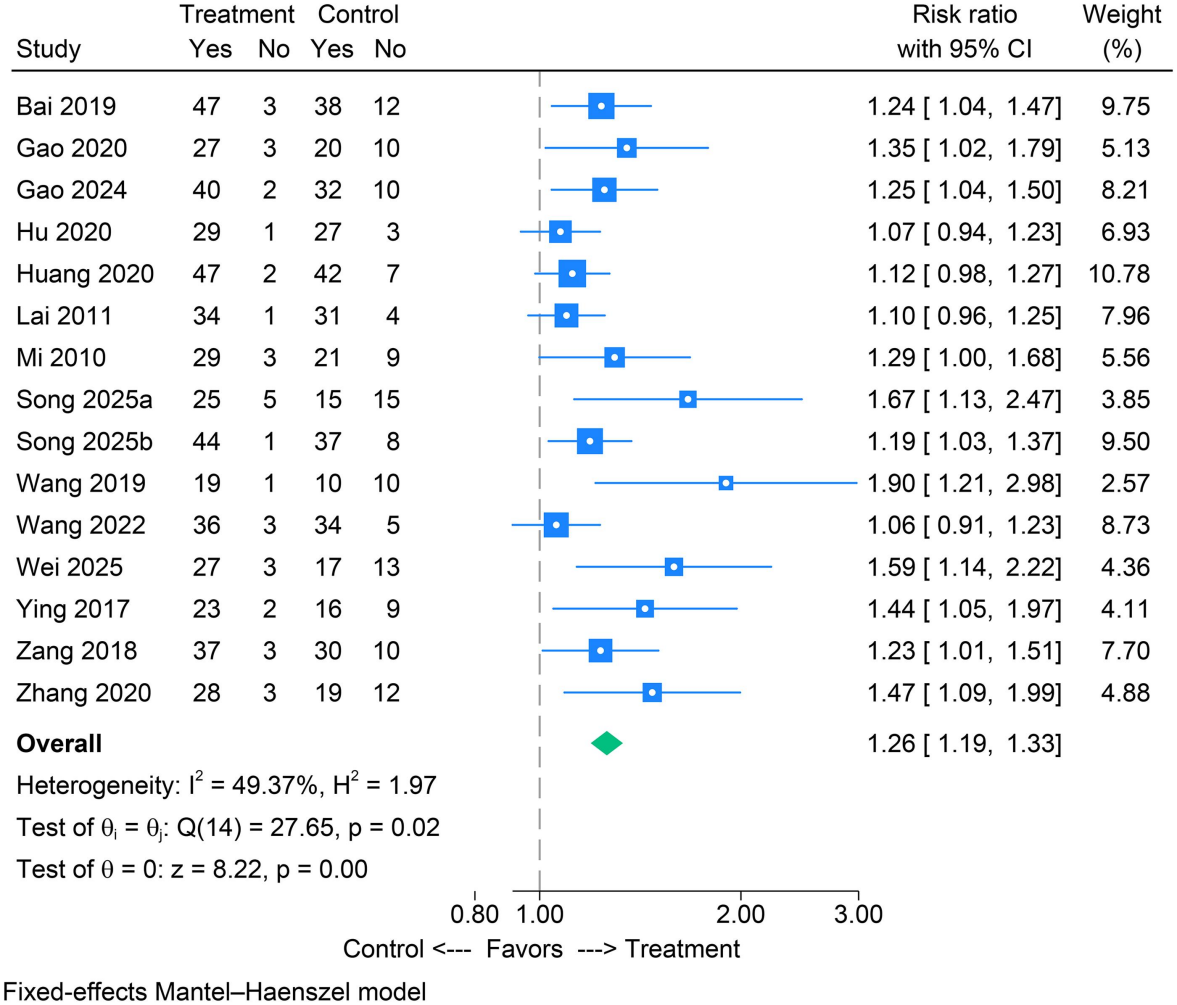
Forest plot of the risk ratio (RR) for pain relief rate.

Fifteen studies involving 1,054 participants reported pain relief rates. As shown in Figure 3, the intervention group had a significant advantage over the control group (RR = 1.26, 95% CI: 1.19–1.33, *I*^2^ = 49.37%, *p* < 0.001). Due to the moderate heterogeneity, we used a fixed-effects model according to the pre-specified strategy. Results suggest a clear advantage for warming-dredging acupuncture in enhancing pain relief.

**Figure 4:**
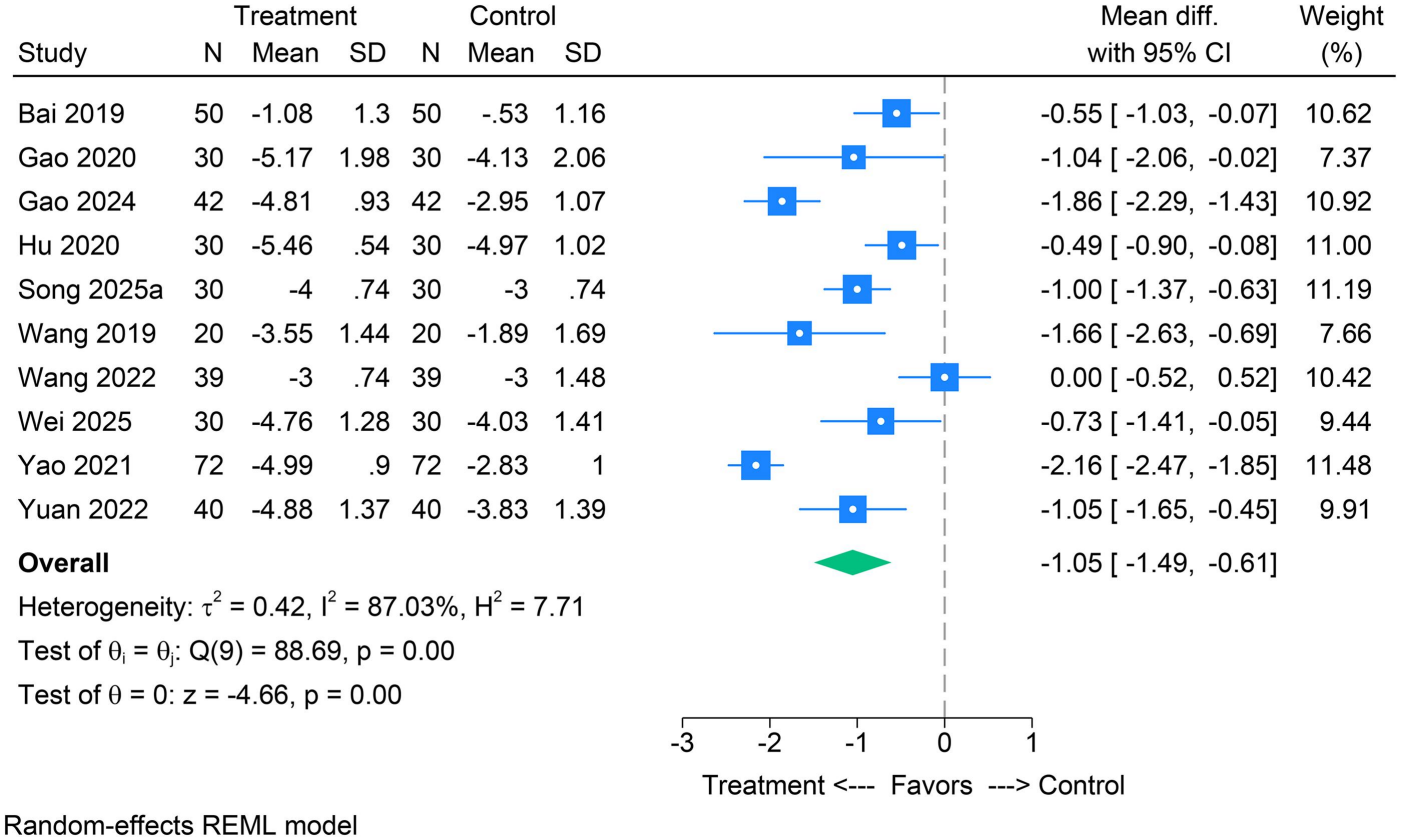
Forest plot of the mean difference (MD) for pain scores.

Ten trials (*n* = 766) documented change from baseline in pain intensity measured by VAS or NRS scores. Figure 4 shows a significant advantage in the treatment group compared with controls (MD = −1.05, 95% CI: −1.49 to −0.61, *I*^2^ = 87.03%, *p* < 0.001). We applied a random-effects model due to substantial heterogeneity. Results indicate that warming-dredging acupuncture could reduce pain intensity.

#### 3.4.2 Subgroup analyses

We performed subgroup analyses to explore the potential sources of heterogeneity and investigate whether specific techniques (fire needle vs. warm needle acupuncture) influenced therapeutic effects. Regarding pain relief rates (Figure S2A), fire needle acupuncture yielded a higher rate (RR = 1.30, 95% CI: 1.19–1.41, *I*^2^ = 31.53%, *p* < 0.001) than warm needle acupuncture (RR = 1.22, 95% CI: 1.14–1.31, *I*^2^ = 58.08%, *p* < 0.001), although the difference was not significant (*p* = 0.31). For changes in pain score from baseline (Figure S2B), warm needle acupuncture appeared to provide greater reduction (MD = −1.39, 95% CI: −2.06 to −0.71, *p* < 0.001) than fire needle acupuncture (MD = −0.70, 95% CI: −1.11 to −0.28, *p* < 0.001). This difference was also not significant (*p* = 0.09). Current evidence does not support a significant clinical difference between these two modalities.

Within the fire needle subgroup, we conducted further grouping by needle types (conventional fire needle vs. filiform fire needle). As shown in Figure S3A, conventional fire needles (RR = 1.39, 95% CI: 1.22–1.57, *I*^2^ = 0%, *p* < 0.001) showed a higher pain relief rate compared with filiform fire needles (RR = 1.20, 95% CI: 1.07–1.33, *I*^2^ = 40.74%, *p* < 0.001), still without a significant difference (*p* = 0.08). Regarding pain scores (Figure S3B), both conventional (1 study) and filiform fire needles (4 studies) demonstrated positive trends in pain score reduction. The conventional fire needles subgroup (MD = −1.00) also showed a non-significant difference (*p* = 0.21) compared to the filiform fire needles subgroup (MD = −0.60). These data do not support that needle types bring different therapeutic effects.

We also stratified the studies by treatment duration (long-term: >2 weeks; short-term: ≤2 weeks). The long-term subgroup (RR = 1.34, 95% CI: 1.19–1.51, *I*^2^ = 69.11%, *p* < 0.001) showed a higher pain relief rate than the short-term subgroup (RR = 1.23, 95% CI: 1.16–1.31, *I*^2^ = 39.14%, *p* < 0.001), with no significant difference (*p* = 0.22) between groups (shown in Figure S4A). Similarly, the long-term subgroup (MD = −1.35, 95% CI: −2.15 to −0.54, *p* < 0.001) yielded a larger reduction in pain score compared to the short-term subgroup (MD = −0.88, 95% CI: −1.39 to −0.37, *p* < 0.001). The difference (*p* = 0.33) between the two subgroups was still not significant (shown in Figure S4B). These findings suggest that treatment durations do not influence clinical effects.

### 3.5 Secondary outcomes

#### 3.5.1 Analgesic consumption

We did not perform a meta-analysis for analgesic consumption due to the high methodological heterogeneity in outcome measures. The outcomes ranged from mean daily dose, total dosage, to final dosage after treatment, and the types of analgesics used varied. Thus, we only performed a qualitative synthesis. As detailed in Table 2, five trials (32, 34, 39, 40, 42) reported a significant reduction in analgesic consumption when clinicians applied warming-dredging acupuncture combined with standard analgesia compared to standard analgesia. The reduction percentage of analgesic consumption ranged from 10.98% to 49.98%. These data suggest that warming-dredging acupuncture may have the potential to reduce analgesic consumption; however, further evidence is needed.

**Table 2:** Qualitative synthesis of opioid consumption outcomes across included studies.

Study	Duration	Outcome measure	T/C (n)	T group Mean ± SD	C group Mean ± SD	Opioid reduction (%)	*p* value
Gao and Zhang, 2020 (32)	4 weeks	Optimal oxycodone dose (mg/d)	18/18	82.22 ± 8.8	125.56 ± 12.81	34.53%	*p* < 0.001
Hu, 2020 (34)	4 weeks	Total oxycodone dose (mg)	30/30	565.42 ± 8.33	736.46 ± 364.36	23.22%	*p* = 0.01
Song, 2025 (39)	2 weeks	Endpoint opioid dose (mg/d)	30/30	19.1 ± 2.37	35.4 ± 2.38	46.05%	*p* = 0.001
Wang, 2019 (40)	4 weeks	Endpoint oxycodone dose (mg/d)	20/20	16.67 ± 7.41	33.33 ± 14.81	49.98%	*p* = 0.006
Wei et al., 2025 (42)	2 weeks	Total opioid consumption (mg)	30/30	981 ± 188.28	1,102 ± 236.61	10.98%	*p* < 0.05

T, treatment; C, control; n, sample size; SD, standard deviation.

The *p*-value was based on the original studies.

#### 3.5.2 Karnofsky performance Status (KPS)

**Figure 5:**
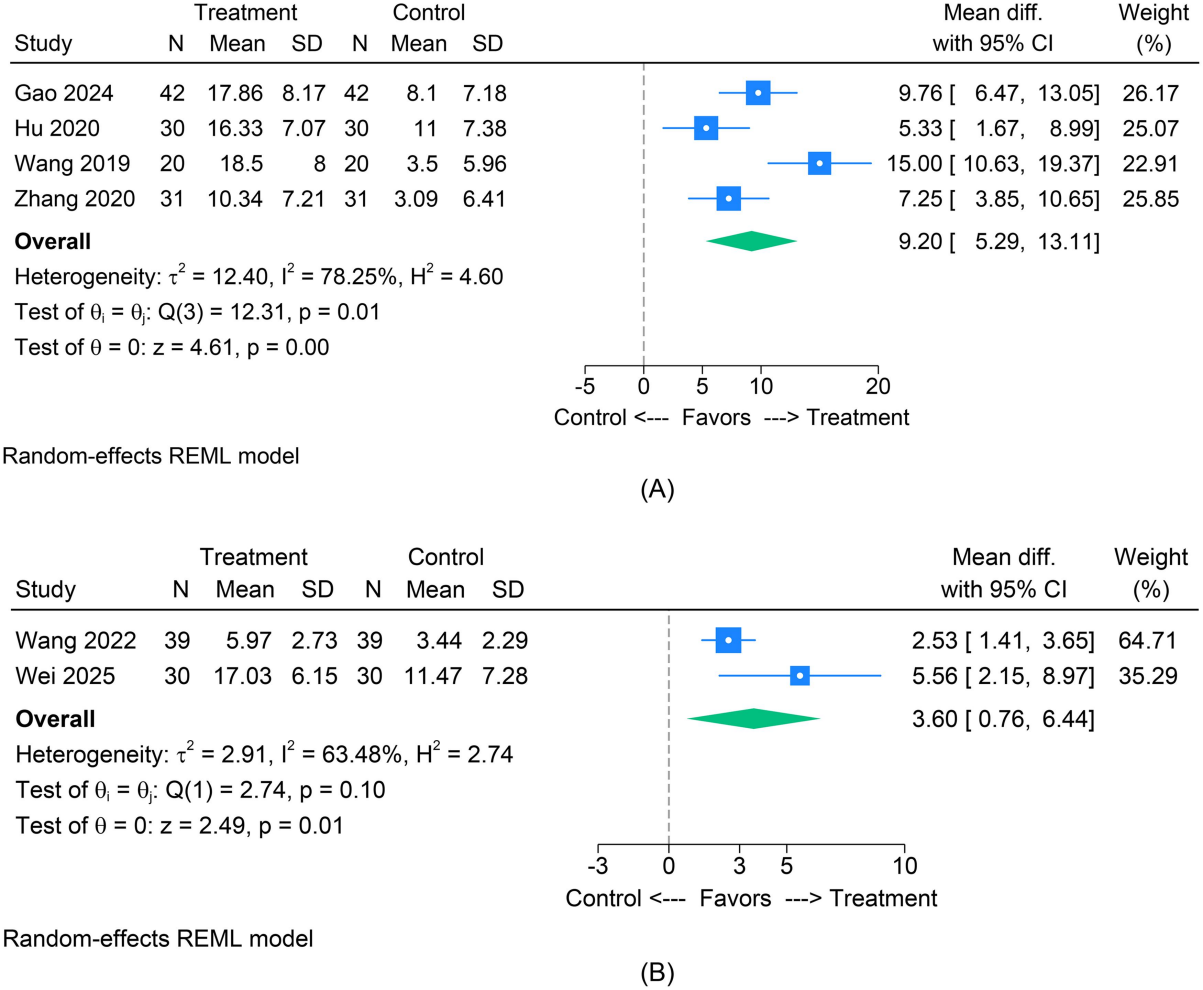
Forest plots for functional status and quality of life: **(A)** karnofsky performance Status (KPS) scores; **(B)** quality of life (QoL) scores.

Only four studies (33, 34, 40, 47) (*n* = 246) reported the KPS data. As shown in Figure 5A, the treatment group had a significant advantage in improving functional status (MD = 9.20; 95% CI: 5.29–13.11; *I*^2^ = 78.25%; *p* < 0.001) compared to the control group. We applied a random-effects model due to considerable heterogeneity. This finding suggests that warming-dredging acupuncture may enhance the performance status of patients with CRP.

#### 3.5.3 Quality of life (QoL)

Six trials (33, 35, 39, 40, 43, 45) reported subscale dimensions (e.g., EORTC QLQ-C30 or General Comfort Questionnaire) without an overall score, which precluded a meta-analysis. Only two studies (*n* = 138) provided analyzable data (41, 42) using the Quality of Life Scale for Cancer Patients (QOL), which is based on a 60-point scale. As shown in Figure 5B, the experimental group showed a statistical advantage (MD = 3.60; 95% CI: 0.76–6.44; *I*^2^ = 63.48%, *p* < 0.001) in QoL scores. This finding suggests warming-dredging acupuncture may improve QoL in patients with cancer pain.

### 3.6 Safety profile

**Figure 6:**
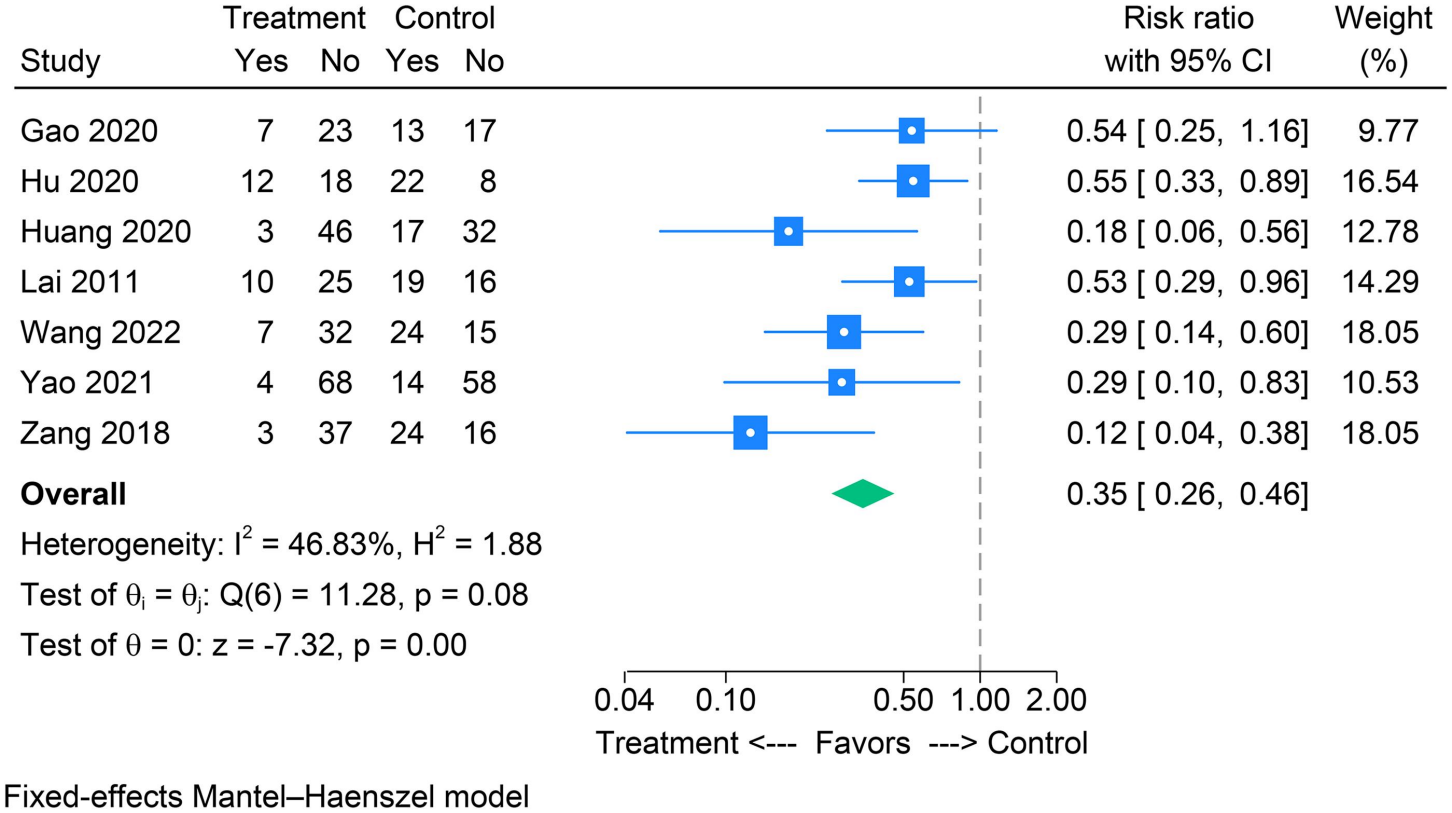
Forest plot of the risk ratio (RR) for adverse events.

Reported adverse events were primarily mild symptoms, such as nausea, constipation, and localized skin hypersensitivity; no serious adverse events occurred. Several studies reported the number of participants experiencing each individual symptom without providing the total number of participants with any adverse events. Seven studies (32, 34–36, 41, 43, 46) (*n* = 590) provided sufficient data for safety meta-analysis. As shown in Figure 6, the experimental group had a significantly lower rate of adverse events (RR = 0.35; 95% CI: 0.26–0.46; *I*^2^ = 46.83%; *p* < 0.001) than the control group. Most reported adverse events were consistent with opioid-related side effects, whereas procedure-related events were not consistently reported. Fewer adverse events were reported in the combined-treatment group than in the routine-analgesia-alone group; however, direct evidence supporting the safety advantage of warming-dredging acupuncture itself was not identified.

### 3.7 Sensitivity analysis

We evaluated whether gray literature (master's theses) influenced the robustness of our primary findings. Thus, we conducted a subgroup analysis. As shown in Figure S5A, the gray literature subgroup (4 studies; RR = 1.27, 95% CI: 1.12–1.43; *p* < 0.001) yielded a similar rate in pain relief compared to the peer-reviewed journal subgroup (11 studies; RR = 1.26, 95% CI: 1.18–1.34; *p* < 0.001). However, the gray literature subgroup showed a considerably higher heterogeneity (*I*^2^ = 81.38% for gray literature vs. 16.23% for journals). But the non-significant intergroup difference (*p* = 0.92) suggests that gray literature did not influence the outcome of pain relief rate. For pain score (VAS/NRS) change from baseline (shown in Figure S5B), the gray literature subgroup (4 studies) yielded an MD of −0.72 (95% CI: −1.33 to −0.10, *I*^2^ = 82.72%; *p* = 0.02), while peer-reviewed journals (6 studies) yielded an MD of −1.27 (95% CI: −1.83 to −0.72, *I*^2^ = 85.44%; *p* < 0.001). The difference between the two subgroups was also not significant (*p* = 0.19), which indicates that gray literature did not significantly affect pain score reduction. Notably, the sensitivity subgroup analysis confirmed that the overall conclusions were not driven by unpublished theses.

We also conducted leave-one-out analyses to verify whether one specific study disproportionately influenced the results for pain relief rate and pain score reduction. The leave-one-out analyses (Supplementary Figures S6a,b) showed that pooled estimates remained stable and within the 95% CIs of the original estimates. Due to the high heterogeneity of pain score reduction (*I*^2^ = 87.03%), we performed a Galbraith plot (Supplementary Figure S7) to explore the source. As shown in Supplementary Figure S7, five studies (31, 33, 34, 41, 43) fell outside the 95% CI region, and one trial (43) showed the farthest distance away from the regression line, appearing to be a primary source of heterogeneity. However, the leave-one-out analysis showed that no single trial determined the overall findings.

For pain relief rate subgroup analyses, we applied fixed-effects models to maintain consistency with the primary analysis. We found that the warm needle acupuncture subgroup and the long-term subgroup showed substantial heterogeneity (*I*^2^ > 50%), so we used random-effects models to verify the results. The reanalysis (shown in Supplementary Figures S8A,B) showed no changes in effect direction, significance, or subgroup differences.

For secondary outcomes, we conducted a leave-one-out analysis of the adverse events (shown in Figures S6C) but did not perform sensitivity analyses for QoL and KPS due to the limited number of studies (*n* ≤ 4), nor for analgesic consumption as a qualitative synthesis without meta-analysis. The leave-one-out analysis of adverse events confirmed the robustness of the pooled effect size and significance.

### 3.8 Publication bias

**Figure 7:**
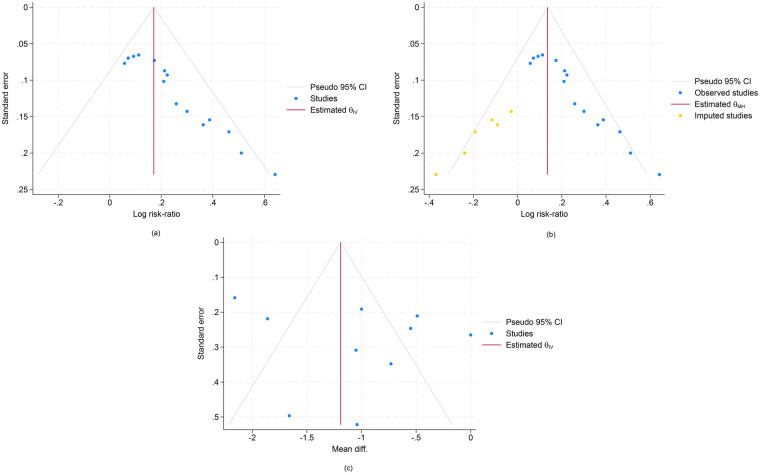
Funnel plots and trim-and-fill analysis for publication bias. **(a)** Funnel plot for pain relief rate; **(b)** trim-and-fill plot for pain relief rate; **(c)** funnel plot for pain score reduction.

Publication bias was assessed by funnel plots and Egger's test when the outcome involved at least 10 trials. Regarding pain relief rate (Figure 7a), the funnel plot (*n* = 15) showed a visual asymmetry, and Egger's test (*z* = 4.37, *p* < 0.001) showed a significant result, which suggested evidence of publication bias or small-study effects. Thus, we applied a trim-and-fill analysis by adding six virtual studies. Although the new funnel plot (Figure 7b) showed a statistically significant effect (RR = 1.15, 95% CI: 1.10–1.20), publication bias or small-study effects may have inflated the primary estimate. For pain score reduction (Figure 7c), the funnel plot was difficult to assess visually for asymmetry; however, the Egger's test (*z* = 0.17, *p* = 0.863) suggested that no evidence of publication bias existed. The number of studies for other outcomes was too small to perform a funnel plot.

### 3.9 GRADE assessment

To assess the certainty of evidence for outcomes, we used the GRADE framework (Table 3). The evidence for adverse events was judged as moderate, and the evidence for pain relief rate and pain score reduction was rated as low. Evidence for KPS, QoL and opioid consumption was rated as very low. The reason for downgrading was outlined as follows:

**Table 3:** Summary of findings (GRADE): warming-dredging acupuncture for cancer-related pain.

Outcomes (post-treatment)	Participants (studies)	Effect size (95% CI)	Certainty of the evidence (GRADE)	Interpretation
Pain relief rate (higher is better)	1,054 (15 RCTs)	RR = 1.26 (1.19–1.33)	⊕⊕◯◯ Low^b^^,^^e^	May enhance pain relief.
Pain score changes (lower is better)	766 (10 RCTs)	MD = −1.05 (−1.49 to −0.61)	⊕⊕◯◯ Low^b^^,^^c^	May reduce pain intensity.
Opioid consumption (lower is better)	298 (5 RCTs)	Not estimable^a^	⊕◯◯◯ Very low^b^^,^^c^^,^^d^	May reduce analgesic dosage; effect size is uncertain.
KPS (higher is better)	246 (4 RCTs)	MD = 9.20 (5.29–13.11)	⊕◯◯◯ Very low^b^^,^^c^^,^^d^	May improve functional status.
QoL (higher is better)	138 (2 RCTs)	MD = 3.60 (0.76–6.44)	⊕◯◯◯ Very low^b^^,^^c^^,^^d^	May improve the quality of life in patients.
Reported adverse events (lower is better)	590 (7 RCTs)	RR = 0.35 (0.26–0.46)	⊕⊕⊕◯ Moderate^b^	Probably associated with fewer reported adverse events in the combined-treatment group.

CI, confidence interval; GRADE, Grading of Recommendations Assessment, Development and Evaluation; KPS, Karnofsky Performance Status; MD, mean difference; QoL, quality of life; RCT, randomized controlled trial; RR, risk ratio.

a Synthesized qualitatively without a meta-analysis due to significant methodological variation.

b Risk of Bias: Downgraded 1 level due to lack of blinding for subjective outcomes.

c Inconsistency: Downgraded 1 level due to substantial and unexplained heterogeneity (*I*^2^ = 63.5% to 87.0%); qualitative assessment used for opioid consumption.

d Imprecision: Downgraded 1 level due to small sample sizes (*n* < 300).

e Publication Bias: Downgraded 1 level due to the trim-and-fill analysis attenuating the estimate from RR = 1.26 to RR = 1.15.

Risk of bias: All trials were rated as high risk by using the RoB 2 tool, due to the challenges in implementing blinding in acupuncture settings and the subjective outcome measurement.

Inconsistency: Substantial heterogeneity (*I*^2^ = 87.03%) in pain score reduction, KPS (*I*^2^ = 78.25%), and QoL (*I*^2^ = 63.48%) was not fully resolved by subgroups or sensitivity analysis. For opioid consumption, a qualitative synthesis was performed as significant methodological variation across the included trials, particularly in the reporting of analgesic dosages and outcome measurement timepoints, precluded a meta-analysis.

Imprecision: The limited number of trials and small sample sizes contributed to the imprecision for KPS (4 studies, *n* = 246), QoL (2 studies, *n* = 138) and opioid consumption (5 studies, *n* = 298).

Publication bias: For the pain relief rate, the funnel plot showed visual asymmetry, and Egger's test was significant. The trim-and-fill analysis attenuated the pooled estimate from RR = 1.26 (95% CI: 1.19–1.33) to RR = 1.15 (95% CI: 1.10–1.20), suggesting publication bias or small-study effects. Therefore, the certainty of evidence for pain relief rate was downgraded for publication bias.

## 4 Discussion

### 4.1 Interpretation of findings

This study was the first systematic evaluation of warming-dredging acupuncture for cancer-related pain, especially in terms of efficacy and safety. The results showed that warming-dredging acupuncture combined with routine pain management was associated with a higher pain relief rate (RR = 1.26) and reduced pain scores from baseline by 1.05 points. The finding supports the potential value of multimodal analgesia. Furthermore, warming-dredging acupuncture combined with routine pain management was associated with fewer reported adverse events (RR = 0.35) and a potential opioid-sparing effect. The improvements in KPS and QoL suggested that warming-dredging acupuncture may contribute to comprehensive clinical benefits beyond pain relief. We used the GRADE assessment to assess the evidence as low grade for both pain relief rate and pain score changes from baseline. The high heterogeneity (*I*^2^ = 87.03%) in pain score reduction, which led to the downgrading of the evidence level, may be due to the variations in malignancy types, tumor stages, and specific acupuncture protocols across trials. However, subgroup and sensitivity analyses generally supported the direction of the analgesic effect. These therapeutic effects may result from the combination of “warming” and “dredging” effects, a mechanistic hypothesis further explored in the following section.

### 4.2 Comparison with previous research

To our knowledge, this is the first meta-analysis specifically evaluating warming-dredging acupuncture for cancer-related pain. Previous studies focused on acupuncture rather than specifically warming-dredging acupuncture. Our findings are broadly consistent with previous reviews (48–50), showing positive effects on pain relief. However, whether warming-dredging acupuncture provides greater benefits than conventional acupuncture requires further evidence from direct comparative studies. Furthermore, while prior research (51) focused primarily on efficacy rather than the safety profile and opioid-sparing effect, our study systematically assessed these critical outcomes. Our review adds evidence for warming-dredging acupuncture, though findings on analgesic use and adverse events are limited by very low certainty and incomplete procedure-related safety data.

### 4.3 Cancer pain management

Our findings suggest that warming-dredging acupuncture may be a useful component within multimodal analgesia frameworks, which are increasingly recommended in clinical practice. The warming-dredging acupuncture group showed a higher pain relief rate (RR = 1.26) than the control group. In clinical practice, pharmacological analgesics are limited by toxicities and reach an efficacy ceiling; this treatment provides an additional adjunctive effect on pain control in this state, offering more choice for both clinicians and patients. Warming-dredging acupuncture appears to offer synergistic efficacy and may address some limitations of pharmacological therapy. The perceptible clinical benefits enhance the confidence of patients in pain control, which improves patients' compliance, thereby fostering a positive therapeutic cycle.

### 4.4 Opioid-sparing effect and safety profile

Although we did not perform a meta-analysis of analgesic consumption due to substantial heterogeneity, the qualitative synthesis of five trials showed significant reductions in analgesic consumption in four studies and a positive trend in the remaining study. These data show a positive trend in analgesic consumption reduction, although the certainty of evidence was very low. If confirmed in future studies, this potential effect may partly explain the lower incidence of dose-related adverse events and help reduce patients' economic burden.

The intervention group showed a significantly lower rate of adverse events (RR = 0.35; moderate certainty). This finding may result from the opioid-sparing effect, as many pharmacological adverse events are dose-dependent. However, procedure-related adverse events caused by needling or thermal stimulation (e.g., needling-site bleeding, burns, local infection) were not consistently reported. Therefore, this result should not be interpreted as direct evidence that warming-dredging acupuncture itself is a safe treatment. More studies reporting procedure-related evidence are needed.

### 4.5 Mechanism

In traditional medicine, cancer pain is viewed as local blood stasis with systemic deficiency. Acupuncture efficacy aims to elicit Deqi, which triggers endogenous regulatory pathways (52). However, achieving this state traditionally necessitates complex manual manipulation. Warming-dredging techniques (fire and warm-needle acupuncture) achieve Deqi by converting manual input into thermal energy (22). This thermal stimulus stabilizes the Deqi response by activating temperature-sensitive ion channels, yielding neural signals that are comparable to, or exceed, those produced by manual technique alone.

From a modern perspective, the induced thermal effect facilitates vasodilation and enhances microcirculation (53), accelerating the clearance of pain-inducing substances such as bradykinin and prostaglandin E₂ (54). Additionally, heat inhibits the release of peripheral pro-inflammatory cytokines—including TNF-α and IL-1β—thereby desensitizing nociceptors to alleviate inflammatory pain (53). These peripheral effects are complemented by activation of the descending inhibitory system, which promotes the release of neurotransmitters such as endogenous opioids and adenosine in the central nervous system.

Contemporary neurobiology further clarifies this integration through the role of TRPV1, a polymodal receptor sensitive to both heat and mechanical force (21, 55, 56). Thermal and mechanical signals, transmitted via A-delta and C fibers, undergo non-linear summation within the spinal dorsal horn, generating a “1 + 1 > 2” analgesic effect (23, 57). This integration likely represents a key mechanism of warming-dredging acupuncture in cancer pain management.

### 4.6 KPS and QoL

Although significant improvements were observed in KPS (MD = 9.20) and QoL (MD = 3.60), the evidence was rated very low, primarily due to the small sample size and substantial heterogeneity. We infer that pain impairs daily functioning, so pain relief can improve physical status and quality of life. However, the very low level of evidence reminds us to take a cautious interpretation.

### 4.7 Clinical implications

Our findings also offer practical guidance for clinical practice. Because subgroup analyses showed no significant differences across modalities and needle types, clinicians may flexibly select from these options based on clinical situations. The lack of a significant difference between long-term and short-term protocols suggests that therapeutic effects may be achieved regardless of the protocol duration, indicating that the thermal-mechanical stimulus may elicit Deqi to achieve pain relief immediately. Thus, clinicians may choose treatment durations flexibly. ST36 (Zusanli), Ashi, LI4 (Hegu), and CV4 (Guanyuan) provide a reliable foundational framework for acupoint selection.

### 4.8 Strengths and limitations

#### 4.8.1 Strengths

This study is the first systematic analysis to integrate evidence on “warming-dredging” acupuncture to evaluate its effect on CRP and the safety profile, adhering to PRISMA 2020 and being registered with PROSPERO (CRD420261335674). Another key strength is that we applied a series of methods (including Galbraith plots, leave-one-out sensitivity analyses, and the trim-and-fill method) to assess the stability of our findings and explore sources of heterogeneity. We also employed the GRADE system to rate the evidence for outcomes, providing a high-level evidence synthesis. Beyond statistical metrics, our study emphasizes clinical relevance. Opioid-sparing effects are also key strengths of our study.

#### 4.8.2 Limitations

We must acknowledge several limitations in our study:

Methodological Bias: According to RoB 2, trials were assessed as having a high risk in the overall assessment. The physical nature of acupuncture with the non-negligible heat stimulation precludes double-blinding, potentially resulting in bias in subjective outcomes such as VAS/NRS. Thus, we rated the evidence on outcomes from very low to moderate using the GRADE tool.

Substantial Heterogeneity: Heterogeneity persisted despite subgroup and sensitivity analyses. We infer that this heterogeneity may reflect real-world clinical variability, including different cancer types, tumor stages, oncology therapies, and other clinical factors. Another limitation is the clinical heterogeneity introduced by pooling fire-needle acupuncture and warm-needle acupuncture in the primary analysis. Although both modalities involve needle-mediated thermal stimulation, they differ in operation, thermal intensity, stimulation duration, and potential action sites. Therefore, the pooled estimate should be interpreted as an overall effect of warming-dredging acupuncture, and the current evidence is insufficient for modality-specific conclusions.

Imprecision and Reporting Bias: The small number of trials and limited sample sizes for KPS, QoL and opioid-sparing effects limited the precision. Because publication bias was detected for pain relief rate, the primary estimate may have been inflated despite remaining statistically significant after trim-and-fill adjustment.

Geographic Constraints: All trials were conducted in China. Although internal consistency is high, global evidence of warming-dredging acupuncture for cancer pain is lacking. Further international multi-center trials are required to verify that this approach works in diverse ethnic groups.

## 5 Conclusions

This study provides the first systematic evidence suggesting that warming-dredging acupuncture may have synergistic and toxicity-reducing value for cancer-related pain. Our findings suggest that this approach may enhance the pain relief rate (RR = 1.26) and reduce pain scores (MD = −1.05). This approach may also have the potential to reduce analgesic consumption. Warming-dredging acupuncture combined with routine pain management was associated with fewer reported adverse events (RR = 0.35). This therapy may also improve KPS and QoL in patients with CRP. Although some outcomes showed heterogeneity, sensitivity, subgroup, and trim-and-fill analyses generally supported the direction of the findings. Overall, warming-dredging acupuncture may offer a promising adjunct for multimodal cancer pain management, but the certainty of evidence varied across outcomes.

## Data Availability

The original contributions presented in the study are included in the article/Supplementary Material, further inquiries can be directed to the corresponding author/s.
