# Supplementary material for: Warming-dredging acupuncture for cancer-related pain: a systematic review and meta-analysis
Source: Front Pain Res (Lausanne). 2026 Jul 30;7:1870255. doi: 10.3389/fpain.2026.1870255 (PMC13469348; doi:10.3389/fpain.2026.1870255)
Supplement: Supplementary file 1 [file Datasheet1.pdf]

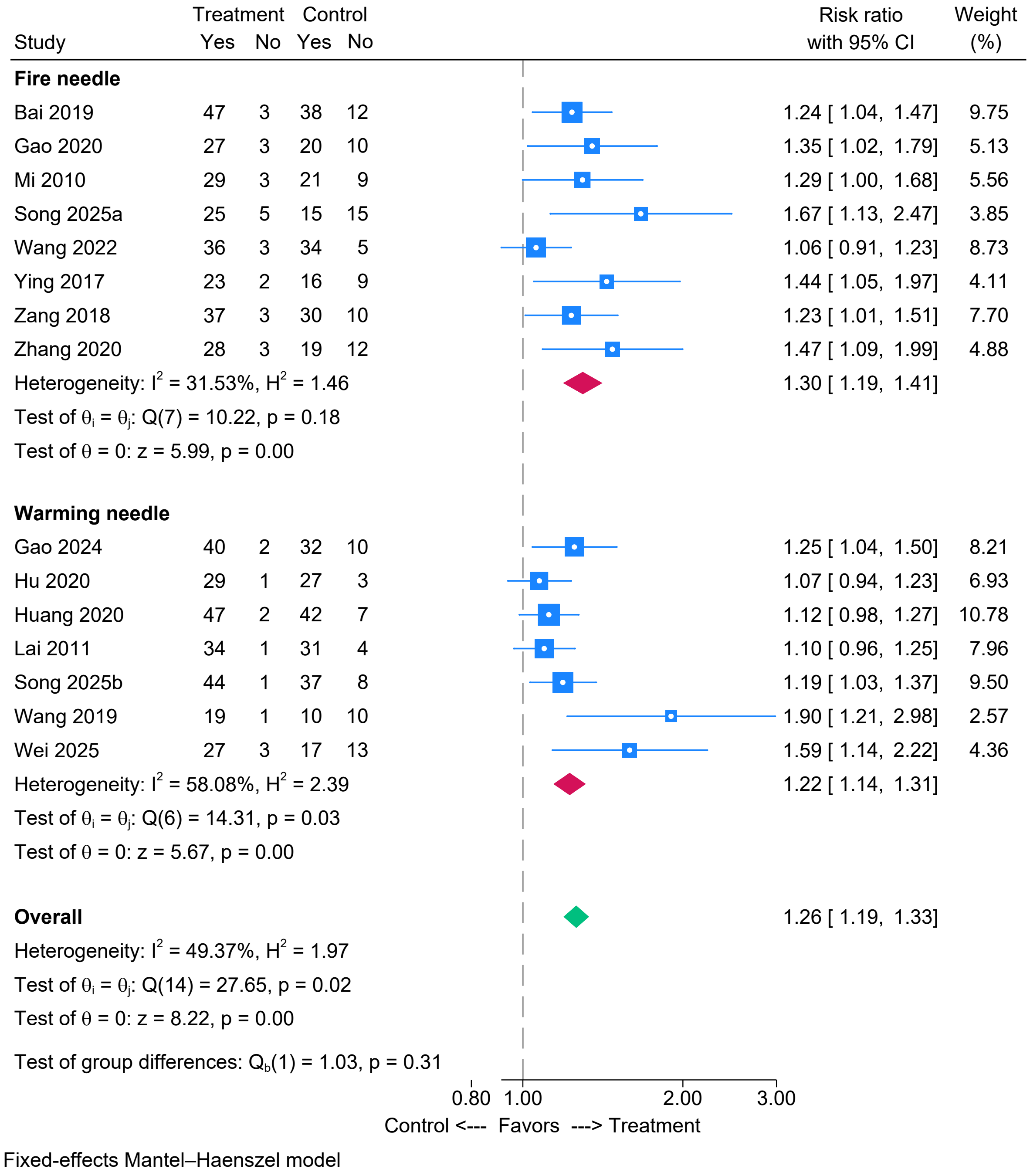

(A)

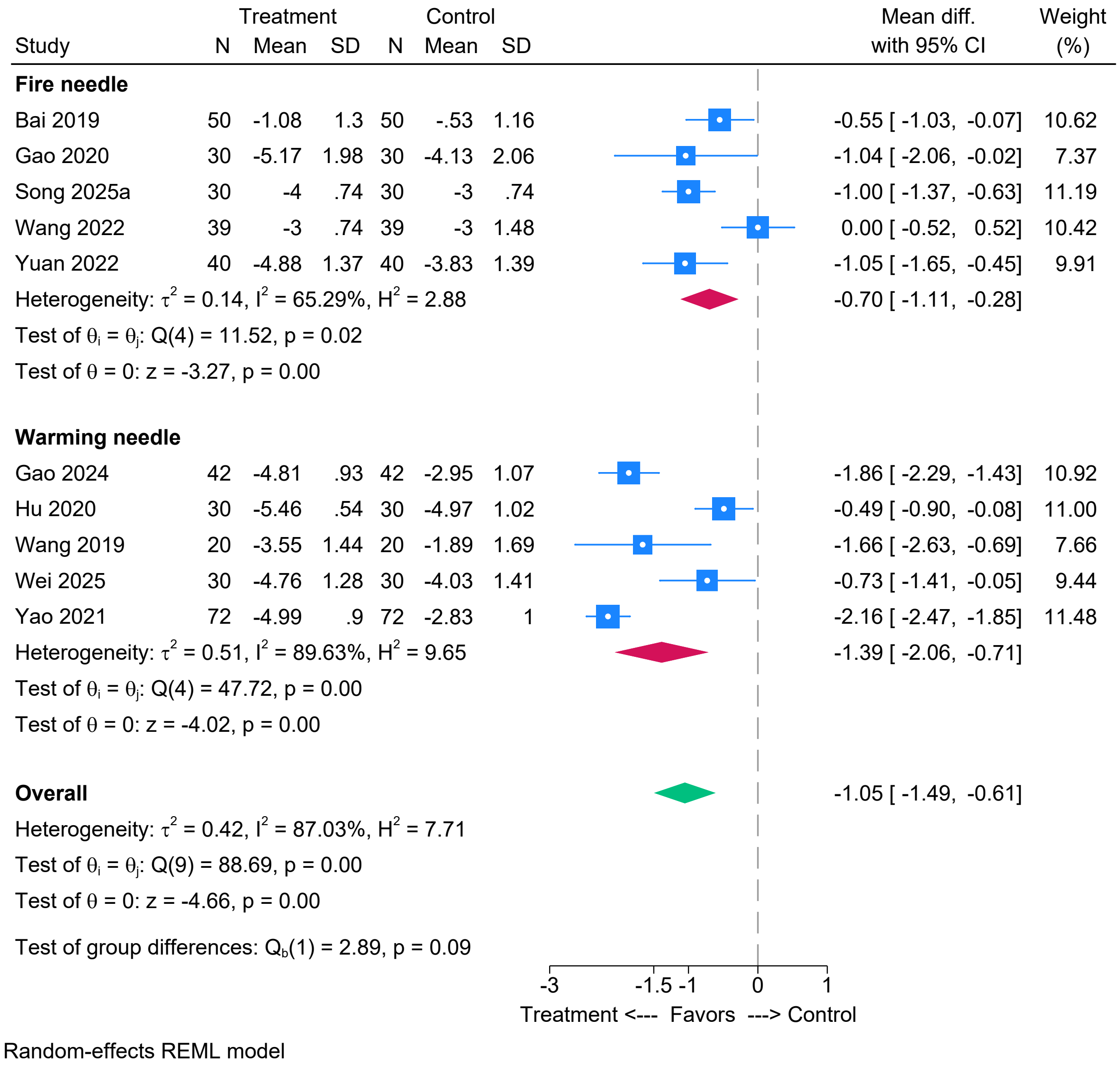

(B)

Figure S2. Subgroup analysis of warming-dredging acupuncture modalities: fire needle vs. warm needle acupuncture. **(A)** Pain relief rate; **(B)** Reduction in pain scores from baseline
