# Supplementary material for: Warming-dredging acupuncture for cancer-related pain: a systematic review and meta-analysis
Source: Front Pain Res (Lausanne). 2026 Jul 30;7:1870255. doi: 10.3389/fpain.2026.1870255 (PMC13469348; doi:10.3389/fpain.2026.1870255)
Supplement: Supplementary file 2 [file Datasheet2.pdf]

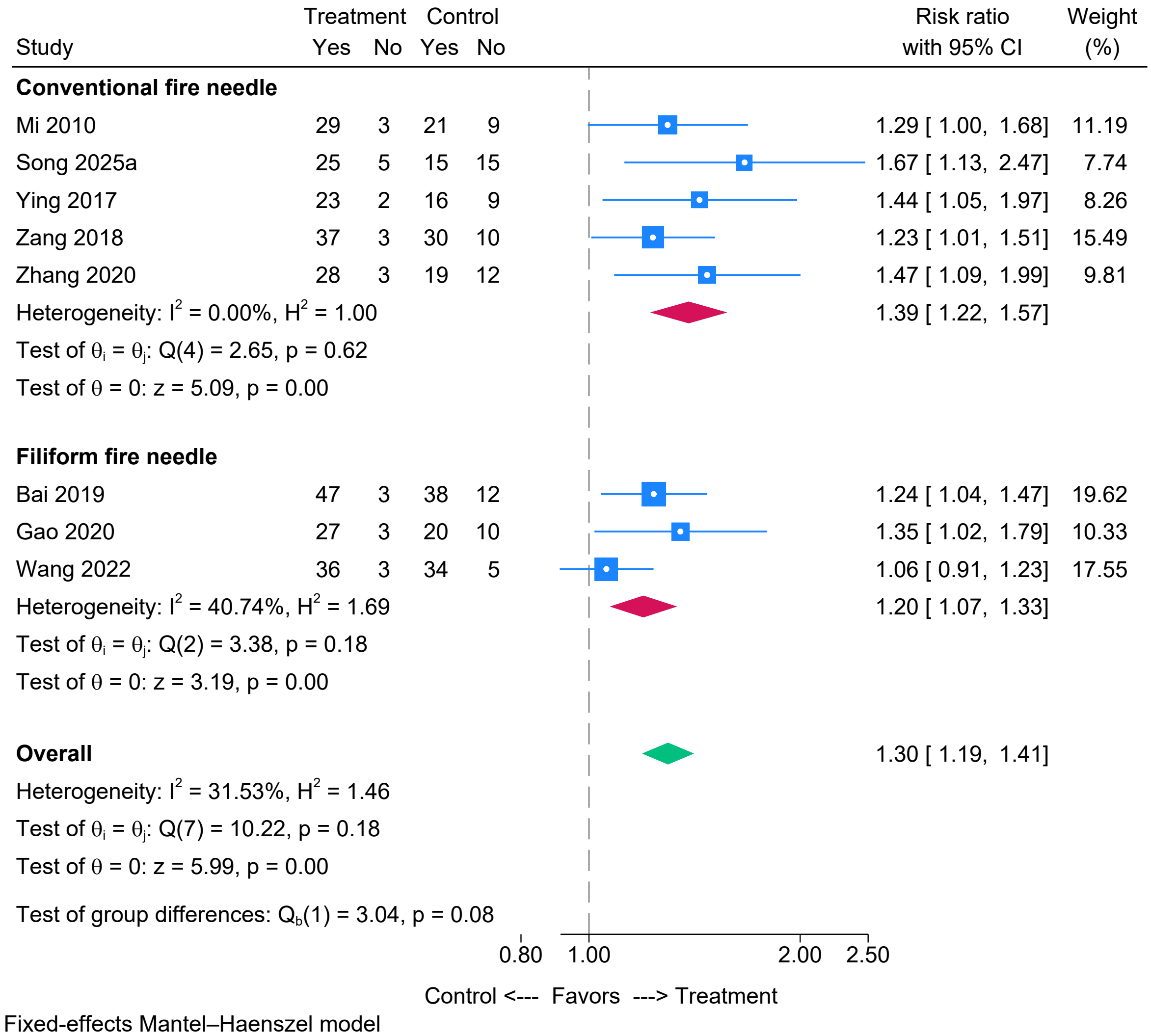

(A)

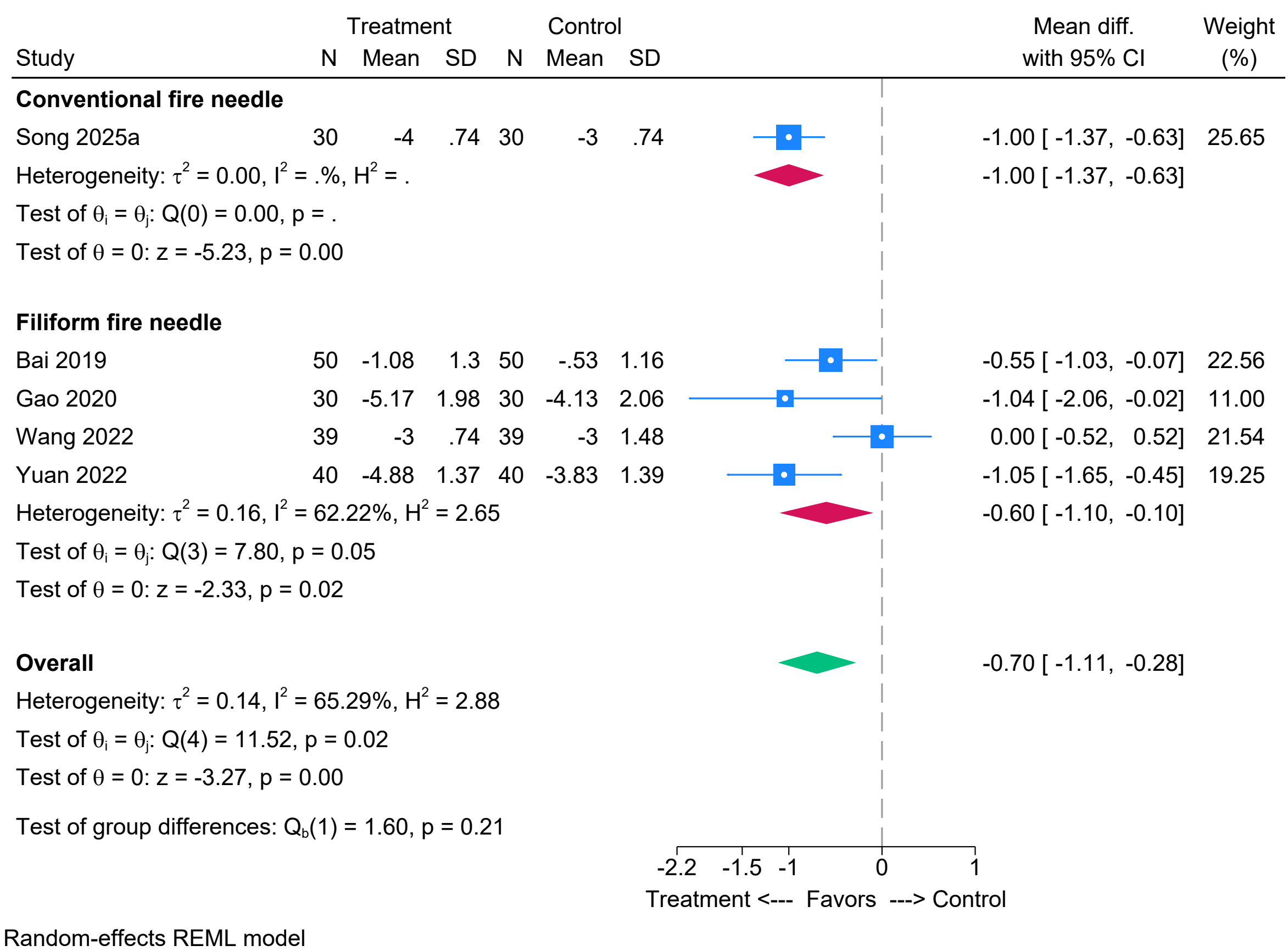

(B)

Figure S3. Subgroup analysis of fire needle types: conventional versus filiform fire needle. **(A)** Pain relief rate; **(B)** Reduction in pain scores from baseline
