# Supplementary material for: Warming-dredging acupuncture for cancer-related pain: a systematic review and meta-analysis
Source: Front Pain Res (Lausanne). 2026 Jul 30;7:1870255. doi: 10.3389/fpain.2026.1870255 (PMC13469348; doi:10.3389/fpain.2026.1870255)
Supplement: Supplementary file 3 [file Datasheet3.pdf]

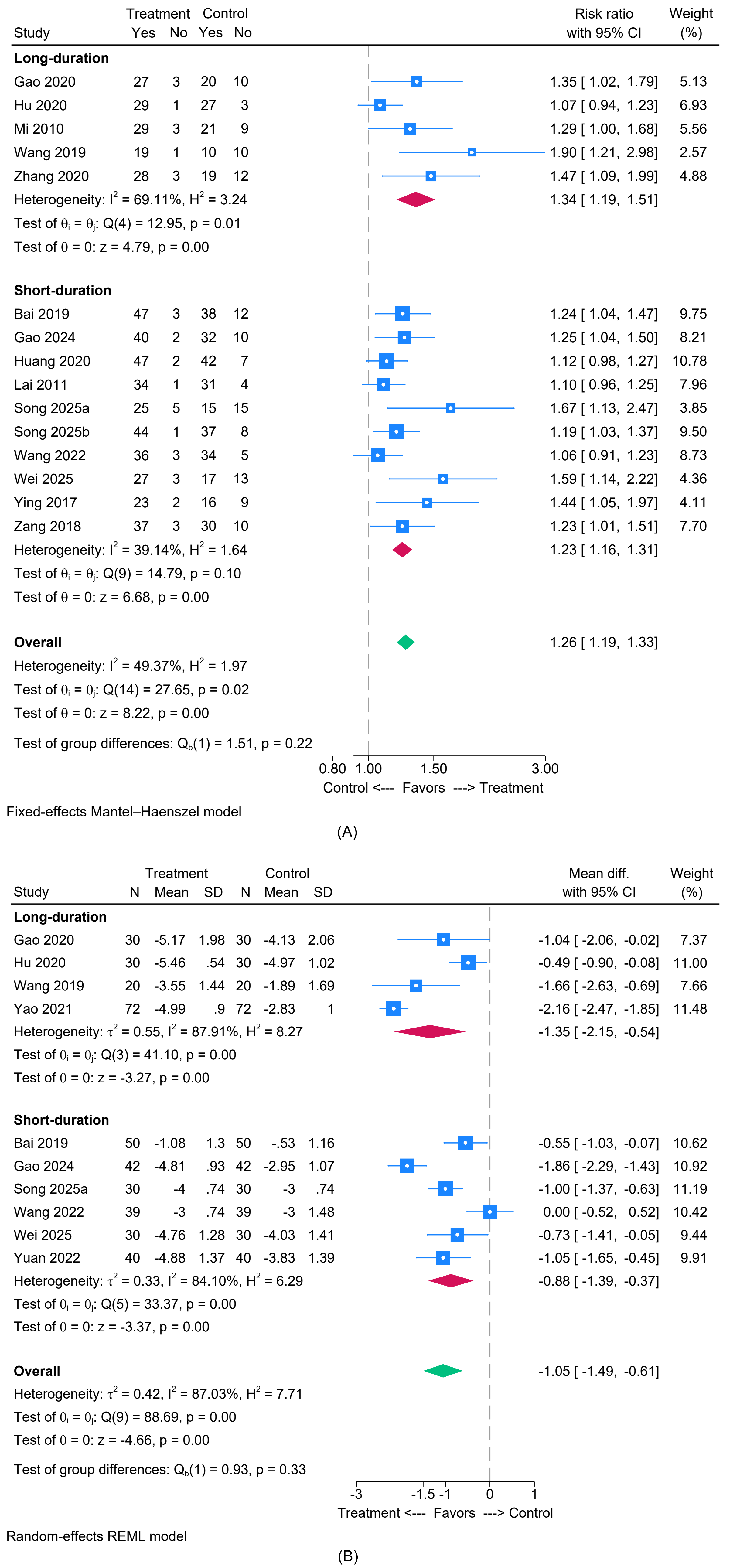

Figure S4. Subgroup analysis of warming-dredging acupuncture by treatment duration. **(A)** Pain relief rate; **(B)** Reduction in pain scores from baseline
