# Supplementary material for: Warming-dredging acupuncture for cancer-related pain: a systematic review and meta-analysis
Source: Front Pain Res (Lausanne). 2026 Jul 30;7:1870255. doi: 10.3389/fpain.2026.1870255 (PMC13469348; doi:10.3389/fpain.2026.1870255)
Supplement: Supplementary file 7 [file Datasheet7.pdf]

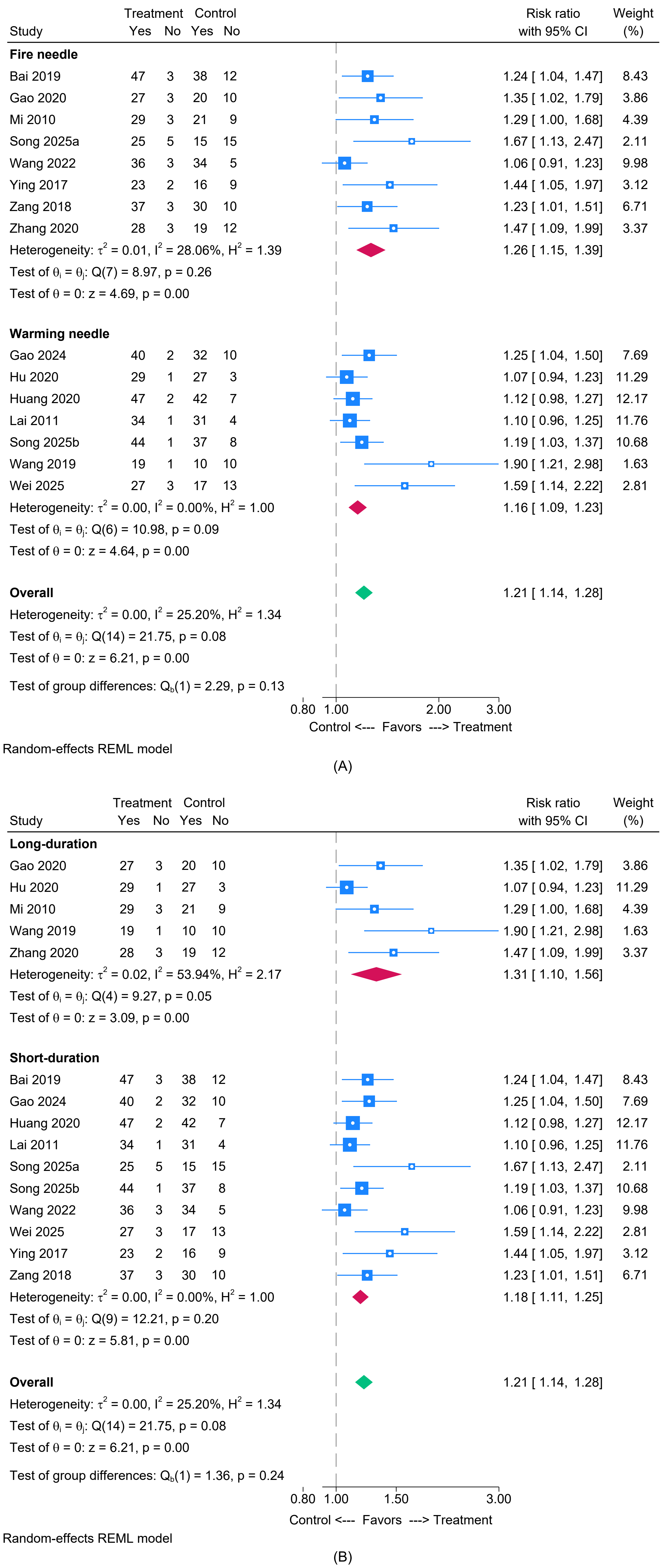

Figure S8. Random-effects re-analysis of pain relief rate. **(A)** By acupuncture modality; **(B)** By treatment duration
