# Supplementary material for: Warming-dredging acupuncture for cancer-related pain: a systematic review and meta-analysis
Source: Front Pain Res (Lausanne). 2026 Jul 30;7:1870255. doi: 10.3389/fpain.2026.1870255 (PMC13469348; doi:10.3389/fpain.2026.1870255)
Supplement: Supplementary file 8 [file Table1.docx]

Supplementary Material

**Table S1.** Search strategies for all databases

| Database | Search Strategy |
| --- | --- |
| PubMed | (((cancer[TW] OR tumor*[TW] OR neoplasm*[TW] OR malignan*[TW] OR oncolog*[TW]) AND pain[TW]) AND ((random*[TW] OR blind*[TW] OR trial*[TW] OR groups[TW]))) AND (("fire needle"[TW] OR "fire needling"[TW] OR (ignited[TW] AND needle[TW]) OR huozhen*[TW] OR "warm needle acupuncture"[TW] OR "warm needling"[TW] OR wenzhenjiu*[TW] OR (needle[TW] AND warming[TW]) OR (moxibustion[TW] AND needle[TW]))) |
| Embase | ( 'cancer pain'/exp OR 'neoplasm pain' OR 'tumor pain' OR 'bone metastasis pain' ) AND ( 'fire needle':ti,ab OR 'fire needling':ti,ab OR 'warm needle':ti,ab OR 'warming needle':ti,ab OR 'needle moxibustion':ti,ab OR 'moxibustion on needle':ti,ab ) AND ( 'randomized controlled trial'/exp OR 'randomization'/exp OR 'double blind procedure'/exp OR 'single blind procedure'/exp OR 'random*':ti,ab OR 'trial':ti,ab ) |
| The Cochrane Library | #1 MeSH descriptor: [Acupuncture Therapy] explode all trees  #2 "fire needle" OR "fire needling" OR "warm needle acupuncture" OR "warming needle" OR "needle moxibustion" OR "moxibustion on needle"  #3 #1 or #2  #4 MeSH descriptor: [Cancer Pain] explode all trees  #5 "cancer pain" OR "malignant pain" OR "neoplasm pain" OR "bone metastasis pain" OR "bone pain" OR "skeletal pain"  #6 #4 or #5  #7 #3 and #6 |
| Web of Science | TS=("cancer pain" OR "malignant pain" OR "neoplasm pain" OR "bone metastasis pain" OR "oncologic pain") AND TS=("fire needle" OR "warm* needle*" OR "warming-dredging" OR "needle moxibustion") AND TS=("randomized controlled trial" OR "randomized" OR "randomly" OR "clinical trial" OR "RCT") |
| CNKI | (SU=('火针'+'焠刺'+'燔针'+'温针灸'+'温针'+'针柄灸')) AND (SU=('癌痛'+'癌性疼痛'+'肿瘤疼痛'+'骨转移')) AND (SU=('随机'+'对照'+'临床研究'))  **English Translation:**  (Subject=('Fire needle' OR 'Ignited needle' OR 'Burning needle' OR 'Warm needle acupuncture' OR 'Warm needling' OR 'Needle handle moxibustion')) AND (Subject=('Cancer pain' OR 'Malignant pain' OR 'Tumor-related pain' OR 'Bone metastasis')) AND (Subject=('Randomized' OR 'Controlled' OR 'Clinical study')) |
| VIP | M=(火针 OR 焠刺 OR 燔针 OR 温针灸 OR 温针 OR 针柄灸) AND M=(癌痛 OR 癌性疼痛 OR 肿瘤疼痛 OR 骨转移) AND M=(随机 OR 对照 OR 临床研究)  **English Translation:**  (Topic=('Fire needle' OR 'Ignited needle' OR 'Burning needle' OR 'Warm needle acupuncture' OR 'Warm needling' OR 'Needle handle moxibustion')) AND (Topic=('Cancer pain' OR 'Malignant pain' OR 'Tumor-related pain' OR 'Bone metastasis')) AND (Topic=('Randomized' OR 'Controlled' OR 'Clinical study')) |
| Wanfang Data | (题名或关键词:(癌痛) or 题名或关键词:(癌性疼痛) or 题名或关键词:(肿瘤疼痛) or 题名或关键词:(骨转移) or 题名或关键词:(骨转移癌痛)) AND (题名或关键词:(火针) or 题名或关键词:(焠刺) or 题名或关键词:(燔针) or 题名或关键词:(温针灸) or 题名或关键词:(温针) or 题名或关键词:(针柄灸)) AND (题名或关键词:(随机) or 题名或关键词:(对照) or 题名或关键词:(临床研究))  **English Translation:**  (Title or keywords: (cancer pain) OR (malignant pain) OR (tumor pain) OR (bone metastasis) OR (bone metastasis cancer pain)) AND (Title or keywords: (fire needle) OR (ignited needle) OR (burning needle) OR (warm needle acupuncture) OR (warm needle) OR (needle handle moxibustion)) AND (Title or keywords: (randomized) OR (controlled) OR (clinical study)) |
| SinoMed | (癌痛 OR 癌性疼痛 OR 肿瘤疼痛 OR 骨转移 OR 骨转移癌痛) AND (火针 OR 焠刺 OR 燔针 OR 温针灸 OR 温针 OR 针柄灸) AND (随机 OR 对照 OR 临床研究)  **English Translation:**  (Cancer pain OR malignant pain OR tumor pain OR bone metastasis OR bone metastasis cancer pain) AND (Fire needle OR ignited needle OR burning needle OR warm needle acupuncture OR warm needle OR needle handle moxibustion) AND (Randomized OR controlled OR clinical study) |
| ClinicalTrials.gov | AREA[ConditionSearch]((cancer pain) OR (cancer-related pain ) OR (malignant pain) OR (neoplasm related pain) OR (tumor-related pain)) AND AREA[InterventionSearch]("Fire Needle" OR "Fire Needling" OR "Warm Needle" OR "Warming Needle" OR "Needle Moxibustion" OR "Warming-dredging") |
| Chinese Clinical Trial Registry | #1,注册题目 (Public Title),癌痛  #2,干预措施 (Intervention),火针  #3,#1 AND #2,(癌痛) AND (火针)  #4,注册题目 (Public Title),癌痛  #5,干预措施 (Intervention),温针灸  #6,#4 AND #5,(癌痛) AND (温针灸)  #7,注册题目 (Public Title),癌痛  #8,干预措施 (Intervention),温针  #9,#7 AND #8,(癌痛) AND (温针)  #10,注册题目 (Public Title),癌痛  #11,干预措施 (Intervention),温通  #12,#10 AND #11,(癌痛) AND (温通)  **English Translation:**  #1, Public Title, Cancer pain  #2, Intervention, Fire needle  #3, #1 AND #2, (Cancer pain) AND (Fire needle)  #4, Public Title, Cancer pain  #5, Intervention, Warm needle acupuncture  #6, #4 AND #5, (Cancer pain) AND (Warm needle acupuncture)  #7, Public Title, Cancer pain  #8, Intervention, Warm needle  #9, #7 AND #8, (Cancer pain) AND (Warm needle)  #10, Public Title, Cancer pain  #11, Intervention, Wen Tong (Warming-dredging)  #12, #10 AND #11, (Cancer pain) AND (Warming-dredging) |
