# Supplementary material for: Warming-dredging acupuncture for cancer-related pain: a systematic review and meta-analysis
Source: Front Pain Res (Lausanne). 2026 Jul 30;7:1870255. doi: 10.3389/fpain.2026.1870255 (PMC13469348; doi:10.3389/fpain.2026.1870255)
Supplement: Supplementary file 9 [file Table2.docx]

Supplementary Material

**Table S2.** Frequency of acupoints used in the included trials.

| Acupoint (Pinyin and Code) | Frequency | Percentage |
| --- | --- | --- |
| Zusanli (ST36) | 10 | 58.80% |
| Ashi points | 6 | 35.30% |
| Hegu (LI4) | 6 | 35.30% |
| Guanyuan (CV4) | 6 | 35.30% |
| Qihai (CV6) | 5 | 29.40% |
| Sanyinjiao (SP6) | 5 | 29.40% |
| Taichong (LR3) | 4 | 23.50% |
| Ganshu (BL18) | 3 | 17.60% |
| Yanglingquan (GB34) | 2 | 11.80% |
| Shenshu (BL23) | 2 | 11.80% |
| Geshu (BL17) | 2 | 11.80% |
| Neiguan (PC6) | 2 | 11.80% |
| Liangqiu (ST34) | 2 | 11.80% |
| Quchi (LI11) | 2 | 11.80% |
| Waiguan (TE5) | 2 | 11.80% |
| Zhongwan (CV12) | 1 | 5.90% |
| Baihui (GV20) | 1 | 5.90% |
| Taixi (KI3) | 1 | 5.90% |
| Fenglong (ST40) | 1 | 5.90% |
| Weishu (BL21) | 1 | 5.90% |
| Tianshu (ST25) | 1 | 5.90% |

Note: ST, Stomach; LI, Large Intestine; CV, Conception Vessel; SP, Spleen; LR, Liver; BL, Bladder; GB, Gallbladder; PC, Pericardium; TE, Triple Energizer; GV, Governing Vessel; KI, Kidney.
